# “We know what we should be eating, but we don’t always do that.” How and why people eat the way they do: a qualitative study with rural australians

**DOI:** 10.1186/s12889-024-18432-x

**Published:** 2024-05-06

**Authors:** Nina Van Dyke, Michael Murphy, Eric J. Drinkwater

**Affiliations:** 1 Mitchell Institute, Victoria University, 300 Queen St, Melbourne, VIC Australia; 2 MM Research, Melbourne, VIC Australia; 3 Centre for Sport Research, School of Exercise & Nutrition Sciences, Deakin University, Geelong, VIC Australia

**Keywords:** Healthy eating, Food choice, Health literacy, Health behaviours, Qualitative methodology, Focus groups

## Abstract

**Background:**

There is evidence that most people are aware of the importance of healthy eating and have a broad understanding regarding types of food that enhance or detract from health. However, greater health literacy does not always result in healthier eating. Andreasen’s Social Marketing Model and Community-Based Social Marketing both posit that, in order to change health behaviours, it is crucial to understand reasons for current behaviours and perceived barriers and benefits to improved behaviours. Limited research has been conducted, however, that explores these issues with general populations. This study aimed to help address this gap in the evidence using a qualitative methodology.

**Methods:**

Three group discussions were conducted with a total of 23 participants: (1) young women aged 18–24 with no children; (2) women aged 35–45 with primary school aged children; and (3) men aged 35–50 living with a partner and with pre- or primary school aged children. The discussions took place in a regional centre of Victoria, Australia. Transcriptions were thematically analysed using an inductive descriptive approach and with reference to a recent integrated framework of food choice that identified five key interrelated determinants: food– internal factors; food– external factors; personal-state factors; cognitive factors; and sociocultural factors.

**Results:**

We found that food choice was complex, with all five determinants evident from the discussions. However, the “Social environment” sub-category of “Food-external factors”, which included family, work, and social structures, and expectations (or perceived expectations) of family members, colleagues, friends, and others, was particularly prominent. Knowledge that one should practice healthy eating, which falls under the “Cognitive factor” category, while seen as an aspiration by most participants, was often viewed as unrealistic, trumped by the need and/or desire for convenience, a combination of Food-external factor: Social environment and Personal-state factor: Psychological components.

**Conclusions:**

We found that decisions regarding what, when, and how much to eat are seen as heavily influenced by factors outside the control of the individual. It appears, therefore, that a key to improving people’s eating behaviours is to make it easy to eat more healthfully, or at least not much harder than eating poorly.

**Supplementary Information:**

The online version contains supplementary material available at 10.1186/s12889-024-18432-x.

## Background

A plethora of recommendations exist regarding how people should eat to maintain better health [e.g., 1–3]. Moreover, there is evidence that most people have a reasonable awareness of connections between healthier foods and better health, and a broad understanding regarding types of food that enhance or detract from health [4–6]. However, greater health literacy does not always result in healthier eating [7–8].

Evidence suggests that public health and health-promotion interventions with a theoretical basis are more effective than those lacking such a foundation [9–11]. Andreasen’s Social Marketing Model [12] posits that a primary focus for behaviour change is on learning what people want and need rather than trying to persuade them to adopt particular behaviours or goals. Community-based social marketing sets out six steps necessary for enacting societal behavioural change; step two is to understand perceived barriers and benefits to develop interventions [13].

Limited research has been conducted, however, that explores how people in the general population eat and their perceptions regarding why they eat the way they do [14–15]. Although several recent papers have examined barriers to and enablers of healthier eating [e.g., 16], relatively few are from the perspective of the consumers themselves [e.g., 17–18] or are narrowly focused on particular types of healthy consumption [e.g., 19].

### Healthy eating: knowing vs. doing

Food-based dietary guidelines are available for more than 90 countries globally. Although there is some variation across guidelines regarding particular foods, there is broad agreement to consume a variety of foods; consume some foods in higher proportion than others; consume fruits, vegetables, and legumes; and to limit sugar, fat, and salt [20–22].

There is mixed evidence regarding whether most people broadly understand what constitutes a healthy diet and believe they should try to eat healthily. A systematic review of the psychological literature on healthy diet, for example, found that the public has a “remarkably accurate” understanding of healthy nutrition and that this understanding reflects key dietary guidelines [23]. Focus groups with participants segmented by age and gender found that most participants were aware of the type of foods that contributed to a healthy diet and the importance of achieving a healthy balance within a diet [24]. Other studies, however, have found evidence of confusion and misperceptions amongst the general public. A cross-sectional survey of 1,097 adults aged 18–64 in Victoria, Australia and 135 professional dietitians, for example, found large discrepancies in which of various food items were considered healthy. Amongst women and those living in higher socio-economic areas, however, views were similar [25]. An earlier survey of Swiss consumers found that between 3% and 38% incorrectly answered procedural nutrition knowledge items. Again, this overall finding differed by sub-groups [26].

However, this knowledge does not necessarily result in healthy eating [27]. A systematic review of the relationship between nutrition knowledge and dietary intake found that the majority of studies reported significant, positive associations, but the relationship was weak (*r* < 0.5*)* and mostly involved slightly higher intake of fruits and vegetables. The authors also noted that study quality ranged widely and that most participants were female and with a tertiary education, with limited representation of individuals from lower socio-economic status background [28]. A qualitative study with adults in New Zealand reported “the impossible rightness of healthy eating”, meaning that the people in their study knew they should be eating healthfully, but simultaneously felt that this was very difficult or impossible to do [29]. A Canadian study argued that the concept of "food literacy" needed to extend beyond nutritional recommendations and cooking lessons to fostering connections between food, people, health, and the environment to bridge this gap between knowing and doing [30].

### Theoretical frameworks

Andreasen’s Social Marketing Model [12] presents behaviour change as the dependent variable, influenced by four classes of independent variables: (1) the attractiveness of behavioural alternatives, (2) community pressures, (3) the cooperation of critical supporting agencies, and (4) marketing efforts. Of specific relevance to this study, Andreasen [12] posits that a primary focus for behaviour change is on learning what people want and need rather than trying to persuade them to adopt particular behaviours or goals.

Also relevant is Community-Based Social Marketing. Community-Based Social Marketing is based on six steps. Step one is to identify the target behaviour– in this case, unhealthy eating. Step two is to understand perceived barriers and benefits to develop interventions [13]. It is this second step that we focus on in this study.

### Food choice

**Fig. 1:**
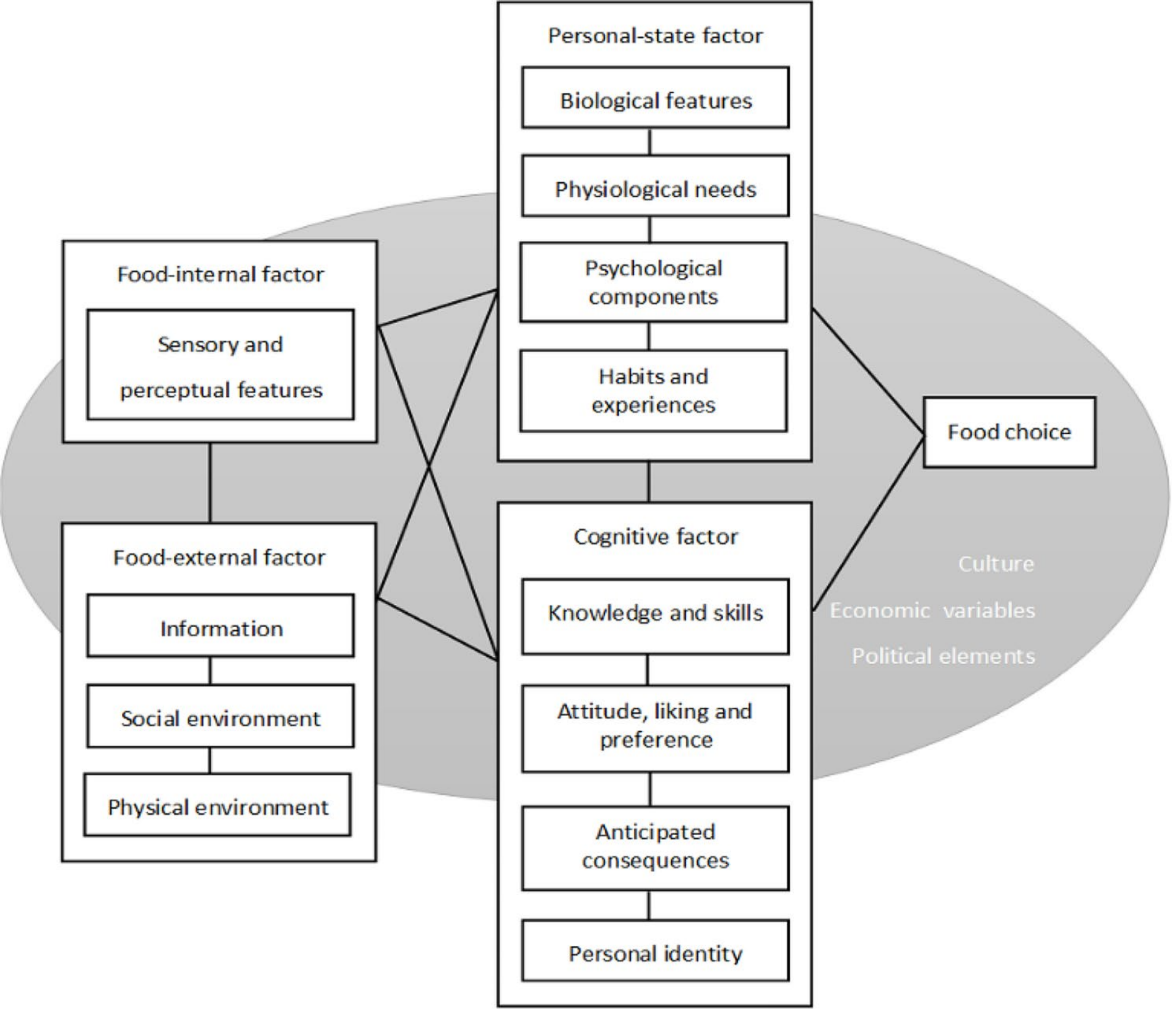
Conceptual model of food choice. The lines in the figure indicate the interactions between different factors [32]

Decisions regarding what food to eat, when, and in what quantity are “frequent, multifaceted, situational, dynamic, and complex” [31]. A recent review and analysis of existing models of food choice integrates key elements into a single framework (Fig. 1) [32]. In this framework, key determinants of general food choice were identified and categorised, including Food– internal factor (sensory and perceptual features), Food– external factor (information, social environment, physical environment), Personal– state factor (biological features and physiological needs, psychological components, habits and experiences), Cognitive factor (knowledge and skills, attitude, liking and preference, anticipated consequences, and personal identity), and Sociocultural factors (culture, economic variables, political elements). According to this framework, any attempt to shift choice must consider these interrelated factors.

### Literature on perceived barriers and enablers of healthy eating

Most of the recent evidence on perceived barriers to and enablers of healthy eating focuses on particular sub-populations, such as young people with obesity, shift workers, or people with Type 2 diabetes [33–37], and/or a particular type of diet, such as the Mediterranean Diet [38–39].

Studies examining more general populations tend to focus on younger people. A scoping review of barriers to and enablers of healthy eating for young adults in Western countries, for example, identified the following barriers: male apathy towards diet; unhealthy diet of friends and family; expected consumption of unhealthy foods in certain situations; relative low cost of unhealthy foods; lack of time to plan, shop, prepare, and cook healthy foods; lack of facilities to prepare, cook and store healthy foods; widespread presence of unhealthy foods; lack of knowledge and skills to plan, shop, prepare, and cook healthy foods; and lack of motivation to eat healthily (including risk-taking behaviour). Key enablers included: female interest in a healthy diet; healthy diet of friends and family; support/encouragement of friends and family to eat healthily; desire for improved health; desire for weight management; desire for improved self-esteem; desire for attractiveness to potential partners and others; possessing autonomous motivation to eat healthily and existence and use of self-regulatory skills [40]. A qualitative study of college students aged 18–24 at one university in Hawaii, U.S., of perceived barriers to and enablers of healthy eating found the largest barriers to be nutrition knowledge deficit, peer pressure, unsupportive institutional environment, and cost. The largest enablers were nutrition knowledge, parental influence, an institutional environment with consistent healthy offerings, and social media. It was noted that several of these factors served as barriers for some participants and enablers for others, such as nutrition knowledge, parental influence, and institutional environment [41]. Another qualitative study with college students at a U.S. college found that common barriers to healthy eating were time constraints, unhealthy snacking, convenience high-calorie food, stress, high prices of healthy food, and easy access to junk food. Conversely, enablers to healthy behaviour were improved food knowledge and education, meal planning, involvement in food preparation, and being physically active. Parental food behaviour and friends’ social pressure were considered to have both positive and negative influences on individual eating habits [42]. Much of this food choice literature identified the importance of social factors and social norms [43–44].

Limited research exists that explores why people in a general population eat the way they do and what, from their perspective, are the barriers and enablers to doing so. From a public health perspective, such evidence is crucial for developing population-level interventions or advocating for policy change. This study aimed to help address this gap in the evidence by using a qualitative methodology to explore the eating patterns and process by which eating decisions were made amongst a general population of non-metropolitan adults in Australia. A non-metropolitan sample was chosen for several reasons. First, Australians living in rural and remote areas experience higher rates of diet-related disease when compared to urban residents, including cardiovascular disease, type 2 diabetes, high blood pressure, chronic kidney disease, and obesity [45–46]. Second, access to healthy food is more challenging in rural and remote Australia due to further distances from urban centres and higher prices [47–48]. Third, Australians living in rural and remote areas experience greater socio-economic disadvantage than those living in urban areas [49], which makes healthy food relatively more unaffordable. Finally, most qualitative research in Australia tends to be conducted with people in metropolitan areas, with less known about people living in non-metropolitan locations.

## Methods

This study is part of a larger, mixed-methods study examining eating behaviours. Data collection took place in 2010. A detailed discussion of the methodology employed for the qualitative component has been published previously in a paper examining what people think of intuitive eating [50]. Other papers published from this study include a quantitative investigation of the associations between intuitive eating and indicators of physical and mental health [51], a review of the literature on the relationship between intuitive eating and health indicators [52], and an experimental study testing whether the accuracy of self-reported height and weight in surveys could be improved by changes to the question wording [53].

### Study design and participants

Three group discussions were conducted with a total of 23 participants: (1) young women aged 18–24 with no children; (2) women aged 35–45 with primary school aged children; and (3) men aged 35–50 living with a partner and with pre- or primary school aged children. These three group demographics were selected to target significant age and life-stages in which shifts in eating behaviours may occur [54]. The groups were conducted in Bendigo, a regional centre of Victoria, Australia, with participants recruited from Bendigo city and outlying areas.

### Procedure

Recruitment was conducted by a professional recruitment agency. Participants were paid AUD70. Participants were chosen such that at least two in each group had previously been on a weight loss diet and at least two had never been on a weight loss diet; at least three in each group were “over my most healthy weight”.

All focus groups were conducted in a hotel conference room facility in Bendigo and were recorded for the purposes of analysis. The groups began with a general discussion about food choices and approaches to eating, including discussion of the factors that influenced food choices. Topics included influences on eating decisions– what, when, how much; eating patterns– when, why, what; feelings around eating; enjoyment of food/eating; and the role that healthy eating played in their decisions around food.

### Data analysis

With the permission of participants, all research sessions were recorded and transcribed. Transcriptions were thematically analysed using an inductive descriptive approach [55–56].

### Ethics

This study received ethics approval from the Charles Sturt University Human Research Ethics Committee (2010/144).

## Results

The conversations about what people ate in terms of choice of food and the amount consumed were contextualised within an appreciation of participants’ living and working situations. While it was beyond the scope of this study to provide a documentation of the diets of participants, some information was provided about specific food preferences. However, the main interest was on the factors that affected their food choices.

Across the groups, there was a general and consistent belief that what one ate was affected by a range of factors, and that as a consequence, none of these participants felt that they were entirely in control of their own diets. While some of these factors were personal, others were felt to be determined by family, work and other social structures.

Participants were clear that the term, “diet”, while most obviously associated with weight loss, can be used to refer to general eating patterns or specific kinds of approaches to eating. Hence, the term, “diet” will be used in this paper to refer to the usual or regular food and patterns of eating. When the topic is related to a specific kind of diet that is being pursued for a particular purpose, this is referred to as the specific kind of diet, and when the specific purpose is related to weight loss, we have referred to this as a “weight loss diet”.

As an inductive approach was used in the analysis, we did not endeavour to match identified themes to the factors presented in the Chen and Antonelli [32] model. Instead, we discuss how our findings align with this model in the Discussion section. Seven main themes were identified, most with several sub-themes. Main themes included taste and health considerations, family factors, work and workplaces, social factors, planning and preparation, meal patterns, and perceptions of own eating.

### Taste and health considerations

Across the groups, participants commonly talked about foods that they liked or did not like and suggested that food tastes and preferences were a primary determinant of their diets. In each group, there was some discussion of eating according to what one feels like at the time. However, it was apparent that this approach tended to mean that people’s eating varied widely in terms of eating healthily or otherwise. While they might experience times when they simply felt like foods that they considered to be healthy, it was apparent that these cravings were not the norm, and that some were almost surprised at the idea of desiring salads or vegetables.

Some days you feel like eating cold meat and salad for tea, or some days you’ll just eat a whole loaf of garlic bread. (Women, 18–24)

Some noted that food preferences seem to go in phases.

I’ve just gone off those. (Women, 18–24)

Participants also commonly talked about health as a factor that would influence their diet, but that they tended to wax and wane in terms of their degree of commitment to maintaining a healthy diet. Even those who reported being quite focussed on health as a motivator felt that it was quite hard to consistently maintain a healthy diet, and that there would be times when they did not feel like making the effort. Underlying these thoughts was a belief that eating healthily was hard work, and certainly harder than eating for convenience.

Mine varies between wanting to be super detox, organic; as natural as possible to, um, I’m totally energy depleted, give me some carbs. So I will, like, live a contradictory diet by having regular meals that are semi-regular, so really, really good, and then just crash and you know you get into work and you come home and you haven’t had time for a proper lunch or you didn’t, you know, take the time to prepare it and they come home after school and… well, it annoys me because I want to be consistent basically, and I want to be role model for my kids as well. (Women, 35–45)

Oh, I have had…I’ll have the healthy breakfast for you know a week or two and then I think, “Oh, I’m sick of that, I’ll just go for toast. You get a bit tired of being strong and healthy. (Women, 35–45)

Some mentioned specific health concerns, including particular diseases or even injuries that affected their capacity to prepare meals.

Oh, our eating habits are very erratic at the moment because I’m not cooking because of an injury, and my husband has to cook so if he’s late home from work, usually the kids have made something for themselves, like a chicken burger or a slice of bread, or a can of spaghetti or something like that. (Women, 35–45)

Within these discussions, it was apparent that participants’ knowledge about nutrition and health varied considerably, and that their level of knowledge did tend to affect food choices. Some participants talked about the idea of balance, and of making choices to ensure a balance of food over the day or week. For some, balance was also about compensating for other aspects of life and health, such as smoking or drinking or physical activity. Some of the men, in particular, talked about doing more activity to compensate for having eaten too much or consumed too much alcohol.

For me, like if I’ve eaten too much, one night I know I’ve got this exercise the next day, so I have to go to the gym or get up in the morning and do some physical activity. (Men, 35–50)

Yeah to me I was the same, I used to smoke and I still drink every now and then you know, I’ll try to keep fit and I know if I eat too much, I’ve got to try and do some exercises to balance it out. (Men, 35–50)

I do heaps of exercise because I love eating… I run so that I can eat. (Men, 35–50)

### Family factors

#### Time and convenience

Throughout the discussions, it was apparent that food choices were substantially affected by factors associated with time and convenience. Participants talked about having busy schedules (e.g., family, work, school, sports), and that these activities had an impact on both the choice and timing of food.

Convenience, especially in terms of the time available for food preparation, was a major factor in food choices. In this context, participants referred to take-away foods, frozen or pre-prepared foods, and meals that were quick to prepare as offering considerable advantage in terms of fitting in with their lifestyles. As noted later, these factors interacted with the time of the week, so that weekdays tended to be more hectic with less time available for food preparation, while weekends commonly afforded greater choice.

#### Household members

Across the groups, participants reported that the choice of food that they consumed at any particular time was not always entirely up to them. Rather, what they ate at any particular meal was commonly affected by where they were eating, who else they were eating with, and other people’s food preferences. This was especially an issue for people who lived with others, most obviously those who were parents and were catering for children and spouses, but also for those who lived in shared households. In this context, the household makeup was a primary determinant of food choices and approaches to eating. This included the mix of males and females in the household as well as the age of children.

That’s me: quick and easy. And I love the chance when I can actually get a recipe, get all the, um, ingredients and make it properly, but that doesn’t happen very often. It’s just usually what’s there and what’s quick. And what everyone will eat. (Women, 35–45)

Oh, yes, that’s a big one for me of having four children and a couple of fussy buggers. You do tend to stick to the things that they will eat… spaghetti bol[ognese], four times a week. (Women, 35–45)

You have to cater for different tastes in the household. (Women, 35–45)

There’s nothing more heartbreaking… when you do go to a lot of effort and they won’t even try it. (Women, 35–45)

In this context, catering for teenage boys was raised as a specific issue. Parents of teenage boys reported that they were often primarily driven by a need to provide filling food, and this tended to mean a reliance on carbohydrate-based meals, such as rice or pasta. Some amongst the group of men also talked about the main motivator for food choices being about filling themselves up. They would choose foods that provided bulk so that they could feel full. Certainly amongst the men, and in the context of parents talking about their sons, there was a substantial focus on the need for food to be bulky and filling.

I usually choose my food for size, value for money and something that the boys will eat. Bigger is better. (Men, 35–50)

Size, you know, steak, parma, my son will eat, you know, most things, money comes into it again, but bigger is better. (Men, 35–50)

I’d rather go big than fancy. (Men, 35–50)

For me I’ve always just, I eat until I’m completely full, if you are breathing and food isn’t coming into your mouth, because you’ve so full, then you are not full enough, so keep eating, that’s the kind of, my whole family is the same, none of them are overweight or fat. (Men, 35–50)

Every second meal is probably pasta or rice [to fill up the kids]. (Women, 35–45)

Throughout these discussions, it was apparent that some of the women who were involved in preparing family meals tended to ignore their own preferences for the sake of catering for partners and children. They believed that it was not worth preparing a different meal for themselves, and so tended to eat whatever they were preparing for others. Several of the women commented that this meant that they did not eat as healthily as they would like to. When prompted, those in the group of mothers commented that they only really enjoyed some of their meals.

Whatever’s in the fridge or cupboard. If there’s salad I’ll have salad, but if we’ve got leftovers I’ll have that… whatever I can grab. (Women, 35–45)

[I enjoy] half to three-quarters [of my meals] and the rest are a bit of a chore. (Women, 35–45)

We’re just eating because you got to eat to keep going, but tea time is more of an enjoyable meal. And the snacks in between are usually enjoyable. (Women, 35–45)

Well, it made me realise that probably maybe it’s more complicated in bringing up children, that I really ignored my own health for quite a long time. (Women, 35–45)

Interestingly, however, some of these same participants commented that when they did have the opportunity to choose meals that were not dependent on the preferences of others, such as when they were at home on their own during the day, they commonly chose foods that were convenient, and reported that they could not be bothered preparing for themselves. They reported that they would find something that they considered simple and easy to make (e.g., leftovers; toast; cheese and biscuits).

Yeah, there are days like that, I just grab one of those [Up & Go drinks]. Um, because I’m part-time sometimes I’ll be home at lunch time and I’ll say to myself in the morning, “Oh, I’ll eat when I go home. I’ll have a good meal when I go home", but what happens is that I stay on at school longer and I’ll come home at 2:00, 2:30/3:00 and then it’s like, “I’ll wait till the kids are home, we’ll just have afternoon…or I’ll come home carb crave, you know, deprived and just…just grab some, like Cruskets or Saladas or some rubbish, a bit of cheese". (Women, 35–45)

*I think if I didn’t have to cook for the kids I would eat differently but, then having said that, as we’ve been talking I thought you know I don’t make the effort at lunch time, I just go by routine, whatever, and…if I’m not enjoying it I’ll just eat it because it’s there rather than spend the time to make something I really like, like vegetables or a salad. A lot of basic things. (Women, 35–45)*

Those who lived with children talked about the age of their children affecting both the kind of food they ate and when they ate. In particular, those with younger children tended to report that they tried to arrange meals around reasonably set timelines. They reasoned that this structure fit in best with other patterns of their children’s day-to-day activities, especially school, sports, and sleep. It was apparent that such set structures were less important for those with older children or without children.

#### Price and budgets

The cost of food was commonly mentioned as a determinant of food choices. This was especially the case for those with teenage boys, given the need to provide large amounts of food. Several of the family participants talked about buying food in bulk when it was cheap and commented that this would then govern their food choices for a period of time.

I buy cereal in boxes of twenty or thirty, so if Nutrigrain is on Special for $4 a box, I buy twenty or thirty… Vita Brits I went and brought, it was $2 a box or something for Vita Brits the other day, and $2 a box for Weet Bix somewhere else, so I actually had a whole car filled with two trolleys full of Vita Brits, Weet Bix, and I haven’t brought Nutrigrain in a while, we are down to about our last three boxes, we had about forty boxes in there the other day. (Men, 35–50)

We’re looking at economy; we’ve all got children. You know, we’ve got to budget. (Women, 35–45)

### Work and workplaces

Outside of the home, some noted that their lunch time food choices when they were at work depended on where they were, what was available, and who else they were eating with or purchasing for. Some commented that they were not always able to take lunch with them to work, and that this, combined with where they were working, determined what they could eat at lunch time. Some commented that they worked in areas with only limited choice and some reported that they would be on the road for work and what they ate depended on which town they were visiting at lunch time. In both of these situations, participants noted that it was especially difficult to make food choices that they believed were healthy, simply because the healthy options were not readily available. Some noted that at their workplaces, a group of workers would take it in turns to decide where they would go for lunch, and therefore the individual’s choice was dependent on what that one place had available that day.

Participants also commented that their workplace, type of work, and working hours determined when they could eat. Some experienced set working hours and had little flexibility to decide when they ate, with references being made to shift work, school hours, or retail businesses with defined customer service hours. Working hours were also regarded as one of the factors that determined whether breakfast was eaten and what was eaten at the time. Some participants talked about not feeling like eating as soon as they got up, preferring to wait until sometime later to have breakfast. However, some of these people also noted that the nature of their work meant that they were unable to eat at the time that they would prefer (e.g., teachers), and therefore that they would have to have something first thing in the morning so they could last through until lunch time.

### Social factors

#### Location of eating

Participants consistently pointed out that eating food that they had not prepared affected their choice of foods, from the perspective of both availability and desire. For example, when eating out, participants reported that they tended to have something they wouldn’t eat at home. They were more likely to have foods they considered to be treats. Some also commented that they would choose foods at these times that were restricted at home because others in the household did not like them. A specific example was food that was provided for free, which was typically at some kind of function. Free food meant different motivations for choice. Partly this was related to not being able to be as fussy as they would be if they were providing their own food or making their own choices. Partly it was related to going for the unusual, commonly more decadent, choice. In both of the above situations (eating out and free food), some participants talked about the idea of feeling like they had to eat all that they were served so as to not waste the opportunity or their money.

Most of the time if I’ve overeaten is when we go to the buffets, where it’s all you can eat sort of thing… so I try to avoid those sort of places, because I will overeat and I feel guilty and then I’ll go out for a walk before I go to bed and then I’ll punish myself the next day. (Men, 35–50)

Other factors related to location were discussed previously under the heading, ‘Work and workplaces’.

#### Social and physical activities

Participants talked about a range of activities that affected both choice and timing of food. A common factor was that of physical activity, and especially in the context of organised team sports. It was noted that these activities, especially if they were during the week, often overlapped with normal eating times, and therefore that meals would need to be rearranged around the activity. With respect to sports, participants also reported that they needed to consider the impact of their meal on their ability to take part in the sport, noting that they might not have sufficient energy to play a sport if they had not eaten, but that they could not eat too soon before being active. This commonly meant that meals on these evenings were either very early or very late, neither of which was regarded as ideal, but something that participants had no control over. It was also noted that physical activity could affect the type of food chosen, specifically that they would need to eat either to provide or replenish energy.

Some of those who were parents also noted that the sports activities of their children affected their own diet, in terms of both timing of meals and choice of food. Because families were reluctant to prepare more than one meal, the whole family had to fit around everyone else’s activities.

Well we have our set days where, like Wednesday nights we have to have Mackie cheese [macaroni cheese] and nuggets, because that’s what the boys want after their swimming lesson, and sometimes I have to go to the supermarket because I haven’t got any left in the fridge, so… pasta is a bit of a staple. (Men, 35–50)

Wednesday is late because I’ve got touch football, so I don’t eat dinner before going to play, I don’t want to go on a full stomach, so lunch is always bigger on a Wednesday than any other day… I hate it because one of the touch footie games isn’t till seven thirty, I hate it, because normally eating at six, there is no way I can have tea beforehand, because I’m just going to run around and get sick, so you don’t get home till… eight thirty, quarter to nine, nine if they are running late, and… yeah, pretty much [McDonald’s] or homemade pizza… because you know they only take about eight minutes in the oven.(Women, 18–24)

Well whether the boys are going to be home or we know they are going to be home or one of the daughters is playing sport or I’m playing sport, it varies. (Men, 35–50)

Participants talked about a range of other social activities, such as various groups and clubs, which affected when and what they ate. While these activities might not have had the same physiological impact on food preferences and choices as sports activities, they did similarly affect when meals were eaten, which in turn affected what was eaten. For example, some mentioned after work activities, which meant that they would not get a chance to eat until late, and by then the quickest and most convenient thing to do was to buy take-away food on the way home or eat pre-prepared frozen meals when they got home.

My partner plays pool on a Monday and Wednesday night, so we always have tea a lot earlier then and cook the simple things that don’t take as long, so he can have dinner before he goes rather than buying pub meals which cost more money.(Women, 18–24)

### Planning and preparation

Throughout the research, it was apparent that different people had different approaches to planning and preparing meals. The approaches tended to depend on factors such as where they lived, how they shopped, and who and how many people they were shopping for. For example, some mentioned that they lived out of town and therefore that they tended to shop less frequently but buy more at a time. Some of those who reported having large families also mentioned that they would shop in bulk. Several of these participants talked about their food shopping being driven by pre-planned meals.

Yeah and as you drift through the town you stop at the supermarket and pick up the required… it’s a half hour drive in and out, so it creates that sense of planning. (Men, 35–50)

For our family… my wife actually sits down each fortnight, because we get paid fortnightly, she works full time, I’m studying full time, and working part time, five kids, the budget is not extensive, so she actually sits down each fortnight and works out what we are going to eat for the fortnight, and then goes and gets all the set ingredients for those meals, and so there’s nothing above and beyond that, now and then there might be a treat thrown in or whatever, all the stuff for the school lunches and that sort of thing. So it’s basically dependent, the amount we eat is dependent on that. She works out ok we need so much to make a meal for seven people. (Men, 35–50)

Participants’ approach to planning was also driven by factors such as their work schedules. They reported that these factors meant that they had different amounts of time available on different days of the week, and therefore that the planning and food preparation process varied according to what was possible on each day.

Oh, well, my aspiration is that I eat more healthily and more natural foods but that’s quite often sabotaged by my planning. My husband probably does want to do that as well but, um, I find it’s often, “Oh, my goodness, I’ve got half an hour to make something and there’s nothing for them, there’s nothing in the fridge, so what are we going to have. So, occasionally it’s fish and chips instead or, um, yeah, just quickly putting something together which isn’t really what I’d want to do but if I’ve done more planning in advance then…(Women, 35–45)

It was also apparent that some participants simply preferred to have a set structure to their diet, and this meant set meals and set shopping patterns.

I guess going back to the getting groceries, I tend to map my weeks out from the Sunday, buy everything for the weekend and that’s it, but I stick to the same recipe every day, so usually lunch is a wrap with ham and a certain amount of grams of tomato and cucumber… it’s just easier to stick to.(Women, 18–24)

I pretty much eat at the same time every day…. 9.30 breakfast, twelve lunch, six o’clock dinner. (Women, 18–24)

By contrast, others tended to be a bit more ad hoc in terms of planning, and therefore shopping. These participants reported that they would decide what to eat each day and might quickly visit the supermarket on the way home. It was apparent during these discussions that this approach was more likely in situations in which men were more involved in day-to-day food choices.

And depending on the timing of the day, what’s happened during the day and that sort of thing, what we feel like, necessarily on the day, will be dependent on… well [my wife] either sorts it out in the morning, or puts the slow cooker on or something like that… [depending on] you know who’s going where, that day, because she’s working, at the moment, she’s teaching up at the uni so she’s there till five o’clock most nights of the week… I’ve got subjects or classes, until four or five, I’ve got one on a Monday that finishes at seven, in the evening. (Men, 35–50)

Finally, participants varied in their attitudes regarding whether they liked to have food in the freezer that could be ready to thaw and prepare, or whether they preferred to buy and eat fresh food.

### Meal patterns

#### Timing of meals

As noted above, participants across these groups reported that their patterns of eating, in particular the time at which they ate, were commonly governed by factors that they felt were external and therefore that they had no control over. Some mentioned that they would eat in the morning because they needed something to get through the start of the day. Even if they did not feel hungry at this time, they were aware that they would feel hungry before there was another chance to eat. From this perspective, for some people and some meals, food was about fuel. They would stock up to prevent themselves running low later on, even if they did not really feel like eating at the time. As noted above, participants in each of the groups talked about the routines and structures of their day-to-day existence determining when they could eat, and that this affected what they would eat. To some extent, they did not feel that this was an ideal approach but felt that they had limited capacity to do otherwise. Hence, in some situations, timing of eating was based on the desire to prevent later hunger, rather than as a response to current hunger.

*I think, I mostly eat because, well I’m hungry and you have to, rather than oh my god that’s fantastic, and I’d love to cook it and eat it and enjoy it, I think it’s just more of a…. (Men, 35–50)*

You’ve got to eat, it’s fuel. (Men, 35–50)

Yeah, like breakfast I wouldn’t normally eat, well I don’t enjoy breakfast, but I eat because I know, come nine o’clock, ten o’clock I’m going to be hungry I’m going to be lethargic, so I’ll force Wheeties in or some toast or… I do enjoy food but I don’t deliberately go out because I enjoy the taste or the texture or whatever, it’s more, well you have to eat. (Men, 35–50)

If I know I’m travelling and I have to skip lunch or something, I’ll probably have a bigger, breakfast than normal, but if I know I’m going to have access to lunch, then no problem, I’ll just have something to keep me, just to get me there, rather than, cook up the big pancakes and the bacon and eggs, you’ve got to taste nice, I’ll be just a couple of bits of toast just to keep the hunger away. (Men, 35–50)

#### Standard and variable meals

Participants were prompted to talk about which meals were standard and which were more variable. For most participants, breakfast, lunch, and dinner were each affected by different factors, as were weekday and weekend meals.

#### Weekday vs. Weekend

Across the groups, weekdays tended to involve more structure, and therefore the weekday meals also tended to involve more structure. This appeared to be most obviously true for those with younger (primary school age) children but was also the case for those with older children and those who did not have or live with children. In other words, the typical weekday involved a degree of externally imposed structure (e.g., working hours: travel times: sporting activities), and for those who lived with others, this was further impacted by the need to coordinate times. For some, food choices tended to be group choices rather than individual choices, especially during the week. By contrast, weekends tended to involve more flexibility of schedules, and as a consequence, more time could be spent in food preparation and decisions about meals were less time and convenience based.

I cook…during the week is when I have…we have set meals and then weekends when I don’t cook… [during the week] we have a meal together every night…at the moment they’re all young so no-one’s out doing things. Yeah, I’m cooking a meal every night, but on the weekend it’s more relaxed, it’s like, “get your own". (Women, 35–45)

#### Breakfast, lunch, and dinner

While there were some exceptions across these groups, breakfast tended to be a more standard and regular meal. To a large degree, this was because time was a major issue, as breakfast needed to be consumed at a set time and in a brief period of time, typically while the family was getting ready for the day’s activities. Interestingly, some participants suggested that they did not experience the same need for variety when it came to breakfast as they did with other meals, commenting that they were happy to have the same thing day after day. As noted above, weekend breakfasts were commonly quite different from weekday breakfasts, being more about choice, enjoyment, and variety than time and convenience. Weekend breakfasts also tended to be more of a family event than simply eating something before the day’s activities.

However, some participants in each of the groups reported that they did not always eat breakfast, typically feeling that it was too early to eat. Amongst this group, some reported having breakfast some days and not others. These people reported they would wake up and decide whether they felt hungry, and if so, what they felt like eating.

It was also common for some to talk about breakfast being a time when they were more in touch with what they felt like eating, or whether they felt like eating at all, although the breakfast choices tended to be quite narrow (e.g., toast: cereal: fruit). Similarly, some reported that they had two or more standard breakfasts, and that they would choose on the day what they “feel like".

I just wake up and whatever I feel like… like if I wake up hungry, then I’ll go and have some, if I feel like cereal, then I’ll have cereal… and if I do sport in the morning, then I usually have toast… I just feel like toast after a run. (Women, 18–24)

It can range from cereal or toast in the morning, my wife makes her own sourdough, so we have that in the morning, which is really good… depends on the mood, because what happens, if the kids wake up, it’s cereal, and I’ll do three bowls at the same time, one, two, three… If everyone is still sleeping, I’ll make my toast and wrap it up and eat it on the way to work so… it just depends on how you feel. (Men, 35–50)

As discussed earlier, lunches tended to vary according to where people were and what they were doing. Convenience was also a key driver for lunch time choices. For those not working during the day, lunches were commonly leftovers from the night before or simple snacks. The mothers talked about not really putting aside time or food for lunch, and often skipping it or simply not getting around to it. If they were not at home, lunch would depend on where they were and what they were doing. For those who were working, there was also the issue of choice being affected by the group, as was previously documented.

Dinner was generally regarded as the most important meal of the day and was afforded more effort and planning. All of the factors discussed previously as influencing food choices tended to be applied to dinners. Most obviously, weekday dinners tended to follow somewhat more of a routine, while there was greater variation and potentially a broader choice on the weekends.

### Perceptions of own eating

Participants were asked to comment on how they felt about their diets and their approach to eating. The typical response was to say that it was mostly okay but could be improved. There was a tendency for participants to comment that they ate too much of some foods that they perceived as not good foods, and/or not enough of other foods that they perceived as good foods. Interestingly though, participants commonly responded to these questions with a range of justifications for the shortcomings that they perceived in their diets. For example, some would claim that it was okay that they ate so much high fat foods because they did a lot of exercise; others would report that it was okay because they had a “good metabolism".

Yeah I’m pretty happy with mine [diet], I think I drink too much Coke, I’m really addicted to Coke, but apart from that I’m pretty happy with it. I really love my vegetables, so we eat a lot of vegies… maybe I do justify it, but I really do think that I eat alright. (Women, 18–24)

I’m so lucky I’ve got a really good metabolism, and also people will be like, I’ve got a block of chocolate down to fifteen minutes, because if I’ve got a five-hour shift, I only get a few minutes, and they are like but that’s so bad for you, yeah but it’s like calcium… and then if I’m at uni and I want to be healthy, I’ll have like steamed dim sims instead of fried dim sims… so I can justify it all in my head, and I know that it’s not right.(Women, 18–24)

Amongst the younger women in particular, some felt that as long as they were happy with their weight, their diet was all right.

Yeah that’s right, I’ll go for a run, and I do exercise, I don’t put on weight, I don’t, but I do exercise, but I think I do justify my bad eating because I don’t put on weight. (Women, 18–24)

Participants were prompted to discuss whether they ever ate too much, and if so, in what circumstances. Generally, participants felt that they were aware when they were eating too much, but as with comments about their diets in general, they tended to have reasons for doing so that made it acceptable in the circumstances. Commonly, participants reported that when they went out for a meal they would clean their plates even if they were full. They reported that serving sizes tended to be large and that they did not want to leave food if they had paid for it. A specific example of this was the ‘All you can eat’ deals. In the context of these discussions, there was some awareness of the idea of stopping before you feel full, but it was apparent that the actual practice of this idea was less than the knowledge. In essence, participants experienced far more benefits to eating till they were full than disadvantages.

A [chicken parmigiana] and a steak and it’s huge, I’ll, because it’s there, I’ll just keep going until it’s finished… half way through I’ve probably had enough, I’ll be thinking I’m not hungry anymore, but I’ll just keep going. (Men, 35–50).

And because you’ve paid for it. (Men, 35–50).

## Discussion

Overall, these findings support Sobal and Bisogni’s [31] contention that food choice is multifaceted, situational, dynamic, and complexx. However, some components of their model received more affirmation than others. A key overarching theme from the findings was the strong and pervasive impact of external forces, or at least the perception of these forces, on what and when food is eaten. Although taste and preferences for particular foods, as well as health considerations, were mentioned, often as competing considerations [57], most of the discussion was about the impact of outside forces on food choice. These included family, work, and social structures, and the expectations (or perceived expectations) of family members, colleagues, friends, and others. According to Chen and Antonelli’s [32] food choice framework, these largely fall into the category, Food-external factors and, in particular, the Social environment sub-category.

The knowledge that one should be practicing healthy eating, which falls under the Framework’s Cognitive factor category, while seen as an aspiration by most participants, was often viewed as unrealistic, trumped by the need and/or desire for convenience, which might be considered a combination of Food-external factor: Social environment and Personal-state factor: Psychological components, in the Framework. Mete et al. [58], in a qualitative study with adults aged 25–58, also concluded that healthy food choices were important but not a daily priority, and that healthy eating information was known but viewed as difficult to apply to everyday life. Other research has noted the importance of convenience in food choice [59–60]. Jabs et al. [61], for example, in a study with low-wage employed mothers, found that most expressed feelings of time scarcity and that, while they prioritised feeding their children, they also wanted to complete meals quickly to move on to other tasks. Bava et al. [62] found that, while the working women in their study said they would ideally choose healthier food, the reality of their lives demanded convenience in food provision to minimise time and cognitive effort.

Other categories and sub-categories of Chen and Antonelli’s [32] framework, while less discussed by participants, were mentioned. Dearth of food choices when travelling for work, for example, might be categorised under Food-external factor: Physical environment. Personal-state factor: Habits and experiences was demonstrated by discussions around eating the same breakfast every day [63]. Personal-state factor: Physiological needs came up in discussions around needing to eat even if one didn’t feel like it in order to not go hungry later in the day, or with men's and boys' needs to eat bulky food to fill up. Desires or cravings for less healthy foods (Food-internal factor) were also perceived as working against the ideal of healthy eating.

Although our study did not seek to explore gender or life stage differences in food choice, several tendencies were observed, which future research may want to further explore. In particular, the women with children discussed food choice largely in terms of what others in the family– i.e., their partner and children– liked and which fit in with their schedules. The men, on the other hand, all of whom had children, more often spoke of eating to fill themselves up, or ‘food as fuel.’ Newcome et al. [64], in a study with partnered men, concluded that men in families displayed unease at expressing enjoyment in food (‘Men downplayed their hedonic consumption’), and instead spoke about food as being largely functional as fuel for their bodies. If these gender and life stage differences prove to be robust, this may suggest quite different public health messaging targeted to women with children, men with children, and those without partners or children. Much of the literature on food choice focuses on women, who continue to be more involved with family food decisions than do their male partners [65], and thus more is known about women’s food choices.

The findings from this study suggest that public health efforts aimed at educating and encouraging individuals to eat more healthfully are, on their own, insufficient to significantly improve healthy eating at a population level. These public health efforts need to be delivered in conjunction with legislation that removes structural barriers to promote healthy eating.

The vast majority of our participants knew they should be eating more healthfully but felt largely unable to do so. Instead, some of these identified structural barriers must be addressed. In particular, improvements to the food environment are needed, particularly in rural areas where distances are greater [66]. Greater provision of quickly preparable, accessible, and reasonably priced food, for example, would assist with some of the time barriers. More workplaces could consider providing free and accessible fruit or other healthy snacks for their employees [67]. Children’s sporting facilities could ensure that healthy foods are available [68].

As with any study, this one has several limitations. First, the focus groups were conducted in 2010; since then, various changes have occurred in the food environment that are potentially relevant to food choice and the findings from this study. These include the rapid proliferation of online food delivery services. There is evidence, for example, that such services increase the geographic access to foods prepared away from home and that these foods tend not to meet healthy eating recommendations [69]. There has also been a significant increase in the production and promotion of convenience and ultra-processed foods over this time [70]. In addition, the marketing of fast food, beverage, and snack brands has expanded via social media [71], with evidence that digital food marketing and social media can influence food choices, preferences, and consumption [72]. Therefore, our findings should be interpreted within this context. Future studies are needed to determine the extent to which the various barriers and enablers to healthy eating identified in this study continue to hold.

Second, the findings of this study are based on only three groups of people with a total of 23 participants, all of whom live in or near a rural region in Victoria, Australia. However, one would assume that many of the discussions around personal, family, and workplace factors would translate beyond this specific group of people, and particularly to other people living in Western countries in non-metropolitan areas. A third limitation of this study is that neither actual dietary intake data nor measures of nutritional knowledge was collected from participants, which would have allowed comparison of what participants discussed against more objective data. However, the focus of this study was on understanding how people think about their eating behaviours and perceptions of motivations and barriers to eating more healthily, rather than on whether their self-reports are factually correct. Moreover, we know that food diary data is often inaccurate [73–74]. Fourth, a single researcher conducted the focus groups and analysed the data. However, with thematic analysis, coding quality is not dependent on multiple coders [75]. The results were discussed with the other co-authors and the first author also read the transcripts. All three authors agreed with the findings.

## Conclusions

Despite a plethora of information regarding how people *should* eat, surprisingly little research explores how and why people eat the way they do– particularly in a general population. Based on findings from focus groups with a range of participants from a rural region of Victoria, Australia, we found that, although decisions regarding when, what, and how much to eat are determined in part by taste preferences and health considerations, they are heavily influenced by a host of other factors. Moreover, many of these factors exist outside the control of the individual, including other household members’ preferences, family activities, and workplace and time constraints, as well as convenience and price. It appears, therefore, that education alone will not solve the problem of unhealthy eating. People want to eat healthier, or at least know they should eat healthier, but it’s all just too hard. It would seem, then, that a key to improving people’s eating behaviours is to make it easy to eat more healthfully, or at least not much harder than eating poorly.

### Electronic supplementary material

Below is the link to the electronic supplementary material.

Supplementary Material 1

## Data Availability

De-identified transcripts will be considered by the corresponding author upon request.Due to the nature of the data (i.e.,dSAZX a small number of focus group participants from a single geographic area), it is very difficult to anonymize the data. In addition, the participants did not provide explicit consent for the transcripts to be shared publicly.

## References

[1] World Health Organization. Healthy diet. World Health Organization. Regional Office for the Eastern Mediterranean; 2019.

[2] Krebs-Smith SM, Pannucci TE, Subar AF, Kirkpatrick SI, Lerman JL, Tooze JA, Wilson MM, Reedy J (2018). Update of the healthy eating index: HEI-2015. J Acad Nutr Dietetics.

[3] Visseren FL, Mach F, Smulders YM, Carballo D, Koskinas KC, Bäck M, Benetos A, Biffi A, Boavida JM, Capodanno D, Cosyns B (2022). 2021 ESC guidelines on cardiovascular disease prevention in clinical practice: developed by the Task Force for cardiovascular disease prevention in clinical practice with representatives of the European Society of Cardiology and 12 medical societies with the special contribution of the European Association of Preventive Cardiology (EAPC). Eur J Prev Cardiol.

[4] Tiedje K, Wieland ML, Meiers SJ, Mohamed AA, Formea CM, Ridgeway JL, Asiedu GB, Boyum G, Weis JA, Nigon JA, Patten CA (2014). A focus group study of healthy eating knowledge, practices, and barriers among adult and adolescent immigrants and refugees in the United States. Int J Behav Nutr Phys Activity.

[5] Manickavasagan A, Al-Mahdouri AA, Al-Mufargi AM, Al-Souti A, Al-Mezeini AS, Essa MM (2014). Healthy eating knowledge among college students in Muscat: a self reported survey. Pakistan J Nutr.

[6] Carrillo E, Varela P, Fiszman S (2012). Influence of nutritional knowledge on the use and interpretation of Spanish nutritional food labels. J Food Sci.

[7] Ross A, Bevans M, Brooks AT, Gibbons S, Wallen GR (2017). Nurses and health-promoting behaviors: knowledge may not translate into self-care. AORN J.

[8] Ronto R, Ball L, Pendergast D, Harris N (2016). Adolescents’ perspectives on food literacy and its impact on their dietary behaviours. Appetite.

[9] Glanz K, Bishop DB (2010). The role of behavioral science theory in development and implementation of public health interventions. Annu Rev Public Health.

[10] Carins JE, Rundle-Thiele SR. Supporting healthy eating behavior through social marketing. Nutrition Science, Marketing Nutrition, Health claims, and Public Policy. Academic; 2023. pp. 231–41.

[11] Harris JA, Carins J, Rundle-Thiele S (2021). Can Social Cognitive Theory Influence Breakfast frequency in an institutional context: a qualitative study. Int J Environ Res Public Health.

[12] Andreasen AR (1995). Marketing social change: changing behavior to promote health, social development, and the environment.

[13] McKenzie-Mohr D, Schultz PW (2014). Choosing effective behavior change tools. Social Mark Q.

[14] Bisogni CA, Connors M, Devine CM, Sobal J (2002). Who we are and how we eat: a qualitative study of identities in food choice. J Nutr Educ Behav.

[15] Monteleone E, Spinelli S, Dinnella C, Endrizzi I, Laureati M, Pagliarini E, Sinesio F, Gasperi F, Torri L, Aprea E, Bailetti LI (2017). Exploring influences on food choice in a large population sample: the Italian taste project. Food Qual Prefer.

[16] Ronto R, Saberi G, Carins J, Papier K, Fox E (2022). Exploring young australians’ understanding of sustainable and healthy diets: a qualitative study. Public Health Nutr.

[17] Rose N, Reeve B, Charlton K (2022). Barriers and enablers for healthy food systems and environments: the role of local governments. Curr Nutr Rep.

[18] Rosewarne E, Chislett WK, McKenzie B, Mhurchu CN, Boelsen-Robinson T, Blake M, Webster J (2022). Understanding enablers and barriers to the implementation of Nutrition standards in publicly funded institutions in Victoria. Nutrients.

[19] Godrich S, Kent K, Murray S, Auckland S, Lo J, Blekkenhorst L, Devine A (2020). Australian consumer perceptions of regionally grown fruits and vegetables: Importance, enablers, and barriers. Int J Environ Res Public Health.

[20] Herforth A, Arimond M, Álvarez-Sánchez C, Coates J, Christianson K, Muehlhoff E (2019). A global review of food-based dietary guidelines. Adv Nutr.

[21] Rong S, Liao Y, Zhou J, Yang W, Yang Y (2021). Comparison of dietary guidelines among 96 countries worldwide. Trends Food Sci Technol.

[22] Fernandez ML, Raheem D, Ramos F, Carrascosa C, Saraiva A, Raposo A (2021). Highlights of current dietary guidelines in five continents. Int J Environ Res Public Health.

[23] de Ridder D, Kroese F, Evers C, Adriaanse M, Gillebaart M (2017). Healthy diet: Health impact, prevalence, correlates, and interventions. Psychol Health.

[24] Chambers S, Lobb A, Butler LT, Traill WB (2008). The influence of age and gender on food choice: a focus group exploration. Int J Consumer Stud.

[25] Niven P, Morley B, Gascoyne C, Dixon H, McAleese A, Martin J, Wakefield M (2022). Differences in healthiness perceptions of food and dietary patterns among the general public and nutrition experts: a cross-sectional online survey. Health Promotion J Australia.

[26] Dickson-Spillmann M, Siegrist M (2011). Consumers’ knowledge of healthy diets and its correlation with dietary behaviour. J Hum Nutr Dietetics.

[27] Spronk I, Kullen C, Burdon C, O’Connor H (2014). Relationship between nutrition knowledge and dietary intake. Br J Nutr.

[28] Guthrie J, Mancino L, Lin CT (2015). Nudging consumers toward better food choices: Policy approaches to changing food consumption behaviors. Psychol Mark.

[29] McDonald A, Braun V (2022). Right, yet impossible? Constructions of healthy eating. SSM-Qualitative Res Health.

[30] Colatruglio S, Slater J. (2014). Food literacy: bridging the gap between food, nutrition and well-being. Sustainable well-being: Concepts, issues, and educational practices, 37–55.

[31] Sobal J, Bisogni CA (2009). Constructing food choice decisions. Ann Behav Med.

[32] Chen PJ, Antonelli M (2020). Conceptual models of food choice: influential factors related to foods, individual differences, and society. Foods.

[33] Brogan E, Rossiter C, Duffield C, Denney-Wilson E (2021). Healthy eating and physical activity among new graduate nurses: a qualitative study of barriers and enablers during their first year of clinical practice. Collegian.

[34] Kebbe M, Damanhoury S, Browne N, Dyson MP, McHugh TL, Ball GD (2017). Barriers to and enablers of healthy lifestyle behaviours in adolescents with obesity: a scoping review and stakeholder consultation. Obes Rev.

[35] Kebbe M, Perez A, Buchholz A, McHugh TL, Scott SD, Richard C, Mohipp C, Dyson MP, Ball GD (2018). Barriers and enablers for adopting lifestyle behavior changes in adolescents with obesity: a multi-centre, qualitative study. PLoS ONE.

[36] Nor NM, Shukri NM, Yassin NQ, Sidek S, Azahari N (2019). Barriers and enablers to make lifestyle changes among type 2 diabetes patients: a review. Sains Malaysiana.

[37] Nicholls R, Perry L, Duffield C, Gallagher R, Pierce H (2017). Barriers and facilitators to healthy eating for nurses in the workplace: an integrative review. J Adv Nurs.

[38] Scannell N, Villani A, Mantzioris E, Swanepoel L (2020). Understanding the self-perceived barriers and enablers toward adopting a Mediterranean diet in Australia: an application of the theory of planned behaviour framework. Int J Environ Res Public Health.

[39] Mayr HL, Kelly JT, Macdonald GA, Russell AW, Hickman IJ (2022). Clinician perspectives of barriers and enablers to implementing the Mediterranean dietary pattern in routine care for coronary heart disease and type 2 diabetes: a qualitative interview study. J Acad Nutr Dietetics.

[40] Munt AE, Partridge SR, Allman-Farinelli M (2017). The barriers and enablers of healthy eating among young adults: a missing piece of the obesity puzzle: a scoping review. Obes Rev.

[41] Amore L, Buchthal OV, Banna JC (2019). Identifying perceived barriers and enablers of healthy eating in college students in Hawai’i: a qualitative study using focus groups. BMC Nutr.

[42] Sogari G, Velez-Argumedo C, Gómez MI, Mora C (2018). College students and eating habits: a study using an ecological model for healthy behavior. Nutrients.

[43] Herman CP, Polivy J, Pliner P, Vartanian LR. Social influences on eating. Volume 5. Cham: Springer; 2019 Sep.

[44] Higgs S (2015). Social norms and their influence on eating behaviours. Appetite.

[45] Australian Institute of Health and Welfare. Rural and Remote Health. Available online: https://www.aihw.gov.au/reports/rural-remote-australians/rural-and-remote-health (accessed on 7 March 2024).

[46] Alston L, Jacobs J, Allender S, Nichols M (2020). A comparison of the modelled impacts on CVD mortality if attainment of public health recommendations was achieved in metropolitan and rural Australia. Public Health Nutr.

[47] Moayyed H, Kelly B, Feng X, Flood V (2017). Is living near healthier food stores associated with better food intake in regional Australia?. Int J Environ Res Public Health.

[48] Whelan J, Millar L, Bell C, Russell C, Grainger F, Allender S, Love P (2018). You can’t find healthy food in the bush: poor accessibility, availability and adequacy of food in rural Australia. Int J Environ Res Public Health.

[49] National Rural Health Alliance. Poverty in rural and remote Australia. Available onlilne: https://www.ruralhealth.org.au/sites/default/files/publications/nrha-factsheet-povertynov2017.pdf (accessed 25 January 2024).

[50] Van Dyke N, Murphy M, Drinkwater EJ (2023). What do people think of intuitive eating? A qualitative exploration with rural australians. PLoS ONE.

[51] Van Dyke N, Drinkwater EJ (2022). Intuitive eating is positively associated with indicators of physical and mental health among rural Australian adults. Aust J Rural Health.

[52] Van Dyke N, Drinkwater EJ (2014). Review article relationships between intuitive eating and health indicators: literature review. Public Health Nutr.

[53] Van Dyke N, Drinkwater EJ, Rachele JN (2022). Improving the accuracy of self-reported height and weight in surveys: an experimental study. BMC Med Res Methodol.

[54] Devine CM, Olson CM (1991). Women’s dietary prevention motives: life stage influences. J Nutr Educ.

[55] Thomas DR (2006). A general inductive approach for analyzing qualitative evaluation data. Am J Evaluation.

[56] Bingham AJ, Witkowsky P. Deductive and inductive approaches to qualitative data analysis. Analyzing and interpreting qualitative data: After the interview. 2021 Apr 8:133– 46. SAGE Publications.

[57] Frank-Podlech S, Watson P, Verhoeven AA, Stegmaier S, Preissl H, de Wit S (2021). Competing influences on healthy food choices: mindsetting versus contextual food cues. Appetite.

[58] Mete R, Curlewis J, Shield A, Murray K, Bacon R, Kellett J (2019). Reframing healthy food choices: a content analysis of Australian healthy eating blogs. BMC Public Health.

[59] Phan UT, Chambers IVE (2016). Motivations for choosing various food groups based on individual foods. Appetite.

[60] Aggarwal A, Rehm CD, Monsivais P, Drewnowski A (2016). Importance of taste, nutrition, cost and convenience in relation to diet quality: evidence of nutrition resilience among US adults using National Health and Nutrition Examination Survey (NHANES) 2007–2010. Prev Med.

[61] Jabs J, Devine CM, Bisogni CA, Farrell TJ, Jastran M, Wethington E (2007). Trying to find the quickest way: employed mothers’ constructions of time for food. J Nutr Educ Behav.

[62] Bava CM, Jaeger SR, Park J (2008). Constraints upon food provisioning practices in ‘busy’women’s lives: Trade-offs which demand convenience. Appetite.

[63] Jastran MM, Bisogni CA, Sobal J, Blake C, Devine CM (2009). Eating routines. Embedded, value based, modifiable, and reflective. Appetite.

[64] Newcombe MA, McCarthy MB, Cronin JM, McCarthy SN (2012). Eat like a man. A social constructionist analysis of the role of food in men’s lives. Appetite.

[65] Daminger A (2019). The cognitive dimension of household labor. Am Sociol Rev.

[66] Lenardson JD, Hansen AY, Hartley D (2015). Rural and remote food environments and obesity. Curr Obes Rep.

[67] Pescud M, Waterworth P, Shilton T, Teal R, Slevin T, Ledger M, Rosenberg M (2016). A healthier workplace? Implementation of fruit boxes in the workplace. Health Educ J.

[68] Kelly B, King L, Bauman AE, Baur LA, Macniven R, Chapman K, Smith BJ (2014). Identifying important and feasible policies and actions for health at community sports clubs: a consensus-generating approach. J Sci Med Sport.

[69] Brar K, Minaker LM (2021). Geographic reach and nutritional quality of foods available from mobile online food delivery service applications: novel opportunities for retail food environment surveillance. BMC Public Health.

[70] Baker P, Machado P, Santos T, Sievert K, Backholer K, Hadjikakou M, Lawrence M. (2020). Ultra-processed foods and the nutrition transition: global, regional and national trends, food systems transformations and political economy drivers. Obes Rev, 21(12), e13126. 10.1111/obr.13126 32761763

[71] Bragg MA, Pageot YK, Amico A, Miller AN, Gasbarre A, Rummo PE, Elbel B. (2020). Fast food, beverage, and snack brands on social media in the United States: an examination of marketing techniques utilized in 2000 brand posts. Pediatr Obes, 15(5), e12606. 10.1111/ijpo.12606 PMC9743983 31875654

[72] Granheim SI, Løvhaug AL, Terragni L, Torheim LE, Thurston M. (2022). Mapping the digital food environment: a systematic scoping review. Obes Rev, 23(1), e13356. 10.1111/obr.13356 34519396

[73] Garden L, Clark H, Whybrow S, Stubbs RJ (2018). Is misreporting of dietary intake by weighed food records or 24-hour recalls food specific?. Eur J Clin Nutr.

[74] Saravia L, Moliterno P, Skapino E, Moreno LA, Food Diary. Food frequency questionnaire, and 24-Hour Dietary Recall. InBasic protocols in Foods and Nutrition 2022 Jun 8 (pp. 223–47). New York, NY: Springer US.

[75] Braun V, Clarke V (2022). Conceptual and design thinking for thematic analysis. Qualitative Psychol.

