# Supplementary material for: “We know what we should be eating, but we don’t always do that.” How and why people eat the way they do: a qualitative study with rural australians
Source: BMC Public Health. 2024 May 6;24:1240. doi: 10.1186/s12889-024-18432-x (PMC11071252; doi:10.1186/s12889-024-18432-x)
Supplement: Supplementary file 1 — Supplementary Material 1 [file 12889_2024_18432_MOESM1_ESM.pdf]

Purpose of the Discussion Guide

This discussion guide has been developed for the use of the group moderator to prompt discussion amongst the participants. This guide will be used to steer discussion to the topic areas to be covered, and the specific questions of interest within each topic area.

For the purposes of qualitative research, it is not necessarily intended that these questions be asked exactly as they are worded here. Focus group discussions are more like a conversation than a set of structured questions. The discussion should be as informal as possible and participants should be encouraged to speak openly and freely. The moderator will need to probe with questions such as "Why?" and "What does that mean to you?" in order to understand participants' responses. The moderator will also need to make sure that all participants in the group have an opportunity to express their opinions.

Because each group of participants will be different, a responsive approach should be used for the research. Therefore, a level of flexibility will be taken in the conduct of each group to allow individual and group reactions to issues For this reason, the groups may vary in terms of the detailed topics and the order in which issues are discussed.

Explanation to participants:

- Introduce Group Moderator.
- Thank participants for their time and contribution.
- Explain what the research is about - "*We're here to talk about issues to do with food and eating.*"
- Explain recording and confidentiality of participant information. – "*With your permission we would like to record the group. The recording will only be used to help us with analysing the results. Your personal details are confidential, and we will not keep or pass on any personal information about you. Is it OK for us to record the group?*"
- Explain the importance of honest opinions – "*Your views and experience are important, so we would like you to tell us what you think and feel about your experiences and about each of the topics we talk about. There are no right or wrong answers to any of the issues we are discussing today, so it is important that you provide us with your honest opinions and that you understand that we will not make any judgements of you for your opinions. Also, as we are talking about your personal opinions and experiences, it is not necessary for everyone to agree with each other. It is helpful for us to find out the different opinions that people have, as well as where people have the same opinions, so please feel free to tell us whatever you think and feel, even if it might be different to what other people in the room are saying*."

Introduction (15 minutes (15))

I'd like to start by asking a bit about yourself: your first name, and a little bit about your home situation, number of kids, work situation, etc.

Eating patterns and eating decisions (25 minutes (40))

- How participants **decide** what, when, and how much to eat
  - How would you describe your approach to eating?
    - What are the main things that influence your eating choices?
    - Prompt re: amount, type, timing, whether they usually eat because they are hungry or for other reasons
    - Prompt re: food available, social context, other ...
  - How do you work out how much to eat at any one time?
    - How does this vary? What factors affect?
    - What do you do if you get served a larger serve than you wanted / needed?
  - I'd like to find out a little bit about everyone's approach to eating. Can we go around the table, and can you each please describe your typical approach to eating?
    - Prompt re food preferences, number of meals, typical meals, etc
    - Is there such a thing as a "normal" pattern for you?
    - What are the things that cause variations to this pattern?
  - Are there meals that you have as standard every day? Which ones?
    - Why do you have those as standard?
    - Why are those ones standard and others more flexible?
      - Probe – is it because of the time of the day, the people you are eating with, the number of people the meal is being prepared for, etc?
    - What is the advantage?
    - What happens if you don't feel like eating your standard meal on occasion?
      - When might that happen?
  - Are there meals that vary every day? Which ones?
    - Why do those particular meals vary?
    - What is the advantage of being able to vary them?
    - How do you work out what to eat on those occasions?
      - What are the things that you take into consideration?
      - Prompt re food available, family preferences, other activities, other ...

Food appreciation (5 minutes (45)) (note: only if time allows and if needed to clarify above questions)

- How participants **feel about the way they eat**
- How much they **enjoy** their meals/snacks/food in general
- How **often** they feel they have **overeaten**/ how do they know / what does this mean
- How participants **feel when** they think they have **overeaten**
  - How do you feel about your approach to eating?
    - Is it the right approach for you?
    - What would you like to change about it? Why? Why haven't you already?
  - To what extent would you say that you enjoy your food?
    - How much / how often do you just eat it because you need to eat?
    - Are there particular times or situations when you don't really enjoy your food? When? Why?
  - Do you ever eat too much? How? Why?
    - How do you tell that you have eaten too much?
    - When do you become aware that you have eaten too much (before, during, after)?
    - How do you feel when you have eaten too much?
      - Specifically, how do you feel about the way you eat at these times?

Dieting (15 minutes (60))

- How participants **feel** about or **experience** they have had with **dieting**
- How much they pay attention to **dieting information** (e.g. fad diets, advertised diets, kilojoules needed to lose weight, have a goal weight)
  - When you say the word 'diet', what do you think of? What does diet mean to you?
  - What has been your experience of weight-loss diets?
  - How do you feel about diets and dieting?
    - Why would you / wouldn't you 'go on a diet'?

    - What are the advantages / disadvantages of ‘dieting’?
    - If you were “on a diet”, what are you hoping to get out of it?
        - Prompt re: weight, clothing size, self esteem, other ...?
- What diets do you know of? (create list)
    - What do you know about each of these?
    - Where do you find out (hear / see / read) about diets?
- How much attention do you pay to all the information out there about diets?
    - What do you specifically listen / look for?
    - What kinds of ideas attract your attention? How do they attract your attention?
    - Prompt for kilojoules / calories, food groups, weight loss, other?
- If you were thinking of a ‘weight loss diet’, what sort of things are important?
    - Prompt re: type of food (carbs, fat, etc); kilojoules / calories; achieving goal weigh / size, etc?
- How do you know which diets are worth trying? Not worth trying?
    - How do you tell what sort of diet is appropriate for you?

Weight (5 minutes (65))

- ➢ Perceived **causes** of **overweight**/obesity
- ➢ Why participants think people living in **rural** areas might have more problems with being **overweight** than those who live in metropolitan areas
    - How would you define ‘overweight’? As far as you know, is there a set definition?
    - How would you define ‘obese’? As far as you know, is there a set definition?
    - How would you define ‘healthy weight?
        - What factors are most important in determining whether a person is overweight?
        - Prompt re: BMI, weight, % body fat, etc?
        - How do you find out this sort of information?
    - As far as you know, what are the causes of overweight?
    - What do you think might be the risk factors for being overweight?
        - In Australia, which groups of people do you think are most at risk of being overweight?
        - Prompt re: sex, age, economic status, location ...?
        - Any thoughts on why greater percentages of people who live in rural areas tend to be overweight or obese?

Intuitive eating (20 minutes (85))

- ➢ Explanation of the **intuitive eating** approach and whether participants do this or have ever done this and what they think about this approach to eating

There are lots of ways to decide when, what, and how much to eat, such as because it’s meal time, the food looks really good, your friends are having it, out of habit, because you’re following a diet, because you’re hungry, etc.

I’m going to explain an approach to eating called ***Intuitive Eating****. The basic idea is that, if listened to, the body inherently “knows” how much and what kinds of food to eat, both to maintain a healthy diet and an appropriate weight. This concept is sometimes referred to as “body wisdom”. There are many things that work to override this innate body wisdom, such as diets, being made to clean one’s plate as a child, eating because it’s “dinner time”, advertisements encouraging people to eat even when they’re not hungry, and so on.*

The fundamental principles of intuitive eating are to regain body wisdom so that you eat only when you’re hungry and stop eating when you’re no longer hungry. There is also no restriction on the types of food you can eat (so, no “good” and “bad” foods) because the body will naturally choose a variety of foods that provide you with nutritional balance.

- What do you think of / feel when you hear this idea of intuitive eating?
- Was there anything in that explanation that you didn’t understand or were unsure of? What?
- What do you like / dislike about the idea?
    - Does this seem like a good way to eat?
    - What seems good / not so good about it?
- How do you think eating this way would affect people?
    - Do you think people would lose weight, gain weight, or stay the same weight if they ate this way?
    - What other ways might it affect them?
- What else do you want to know about it? What questions do you have?
- Do you think you could do it?
    - Why / why not?
    - What would be easy / hard about doing this?

Sum up (5 minutes (90))

- Do you have anything else to say about any of today's topics?
- What has been the most interesting / informative element of this conversation for you?
